# Fraser Syndrome-Oral Manifestations and a Dental Care Protocol

**DOI:** 10.1155/2014/486108

**Published:** 2014-12-21

**Authors:** Talita Lopes de Oliveira, Giselle Rodrigues de Sant'Anna

**Affiliations:** ^1^Cruzeiro do Sul University (UNICSUL), Rua José Garcia de Souza 422, 08673-040 Suzano, SP, Brazil; ^2^Cruzeiro do Sul University, Rua Saturnino dos Santos 106, 04124-150 São Paulo, SP, Brazil

## Abstract

Fraser syndrome is a rare genetic malformation with an autosomal recessive pattern of inheritance and an incidence of consanguinity ranging from 15% to 25%. A 5-year-old male patient who is a carrier of Fraser syndrome initiated treatment in the pediatric dentistry sector. The patient was fed parenterally since birth, experienced recurring bouts of chronic lung disease, and was referred to the pediatric dentistry sector by the medical team. Radiographic examination revealed the presence of all permanent teeth. Supragingival dental calculus, halitosis, and gingival inflammation were also observed. Dental calculus was removed by manual scraping, and chemotherapeutic agents were used, chlorhexidine 0,12%, chlorhexidine gel 2%, and PVP-I, to control the bacterial flora. The patient is still being monitored after an 8-year follow-up period, the complications associated with periodontal disease decreased, and since the initiation of treatment, the patient has not needed to be hospitalized because of chronic lung disease. This study reports the case of a patient diagnosed with Fraser syndrome and describes the clinical manifestations (general and oral).

## 1. Introduction

Fraser syndrome is a rare genetic malformation with an autosomal recessive pattern of inheritance and an incidence of consanguinity ranging from 15% [1] to 25% [2]. This survival rate is very low, and the patients most affected die before reaching one year of age [3]. Fraser syndrome is characterized by multiple malformations, including cryptophthalmia, syndactyly of the hands and feet, and genital anomalies [4] and can be associated with malformations in the kidneys, ear, nose, larynx, and skeleton. One of the main diagnostic features of Fraser syndrome is cryptophthalmia, but this complication is not necessarily associated with the syndrome. George Fraser was the first researcher to diagnose cryptophthalmia associated with multiple abnormalities in two siblings in 1962.

The literature review indicates that orofacial anomalies are rarely reported for Fraser syndrome. These anomalies and their respective current incidence are facial asymmetry in 10% of the cases [5], lip and palate cleft in 2%–11% of the cases, ogival palate in 12% of the cases [2], ankyloglossia [6–9], dental crowding [7, 10], fusion of deciduous teeth [11, 12], and dental hypoplasia [1].

This study reports clinical findings in a patient diagnosed with Fraser syndrome, his general and oral clinical manifestations, and the establishment of a protocol using chemotherapy associated with the manual removal of tartar.

## 2. Case Report

This paper was submitted to the Ethics Committee of Cruzeiro do Sul University with protocol number 969/082014.

A 5-year-old male patient who is a carrier of Fraser syndrome (initials: T. F. O.) initiated treatment in the pediatric dentistry sector at the Secretariat of Health of Barueri, São Paulo, Brazil. The patient is still being monitored after an 8-year follow-up period.

**Figure 1:**
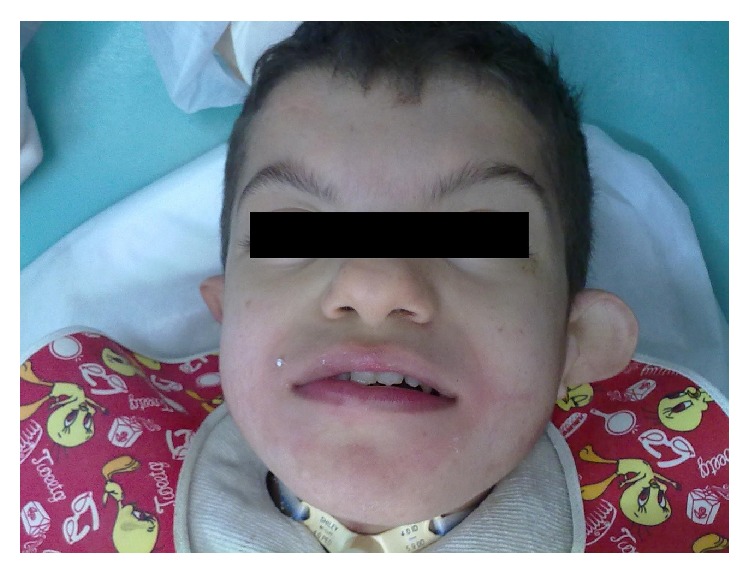
Facial characteristics of the patient: facial asymmetry and bilateral malformations of the ear.

The patient exhibited no cryptophthalmia but presented with facial asymmetry, bilateral malformation of the ear, hearing loss (Figure 1), agenesis of the right kidney, nail clubbing, cryptorchidism, paralysis of the esophagus and trachea, umbilical hernia, laryngotracheomalacia, genital anomalies, and recurrent bouts of chronic lung disease; the patient was fed parenterally from birth.

T. F. O. and his family attended the Basic Health Unit in Barueri in 2000. The patient was referred for a dental examination as a result of recurrent episodes of chronic lung disease with successive hospitalization due to syndrome-related complications. Thus, the patient needed dental and medical assistance by a multidisciplinary team to reduce such complications.

During the interview, the mother denied consanguinity with her husband and reported having a younger child who was not affected by the syndrome. Antibiotic therapy has been a viable intervention for pulmonary prophylaxis. During the first 4 years of treatment, the patient did not establish any form of contact with the oral health team because he had serious mental retardation and consequently did not receive any targeted therapy during this period.

Approximately 3 years after the initiation of dental treatment, the patient was able to establish a relationship with the professional staff, became part of the patient group, and was aware of the physical space where the consultations were being made.

**Figure 2:**
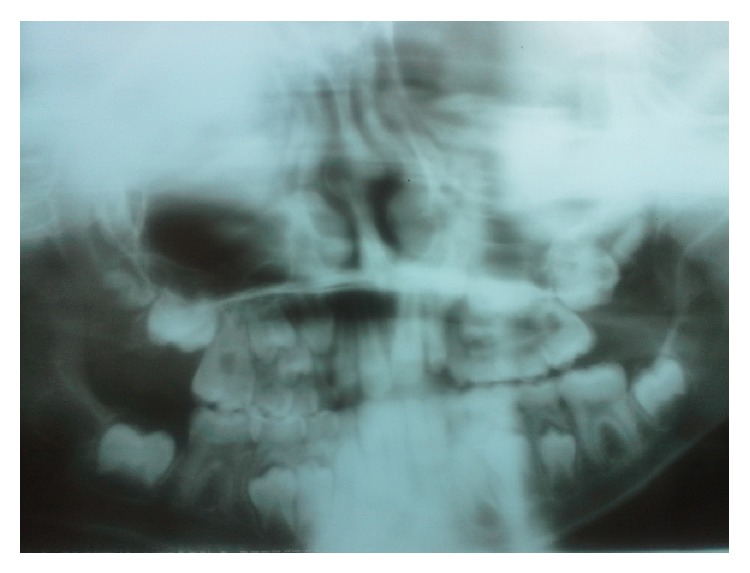
Radiographic examination.

During the intraoral evaluation period, the patient was at the mixed dentition stage, and radiographic examination (Figure 2) revealed the presence of all permanent teeth and no impairment of the periodontal support; that is, the cement, ligament, and bones were intact, despite the presence of marginal gingivitis.

**Figure 3:**
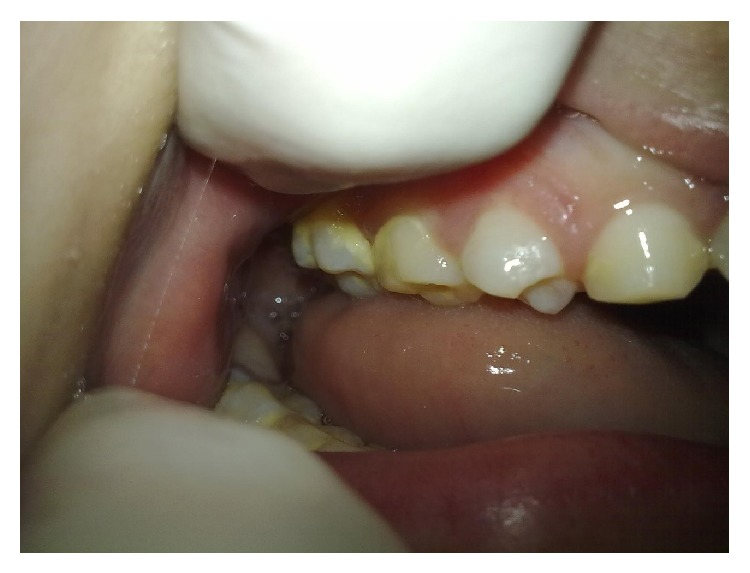
Oral manifestations: presence of dental calculus.

The tooth surface exhibited biofilm, and the presence of supragingival dental calculus was noted primarily in the upper and lower posterior regions and in the lower anterior region (Figure 3).

**Figure 4:**
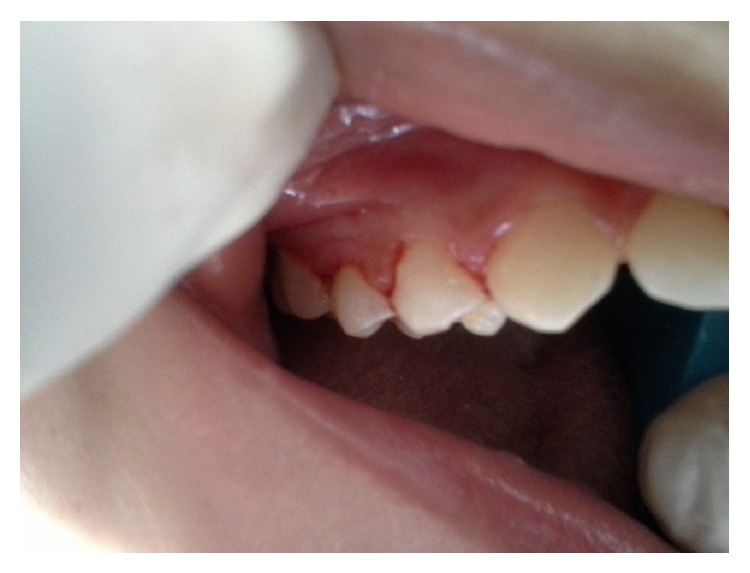
Oral manifestations after treatment.

When establishing an assistance protocol, our first concern was to help decrease the recurrent bouts of pneumonia, and for this reason, the protocol involved the use of chemotherapeutic agents combined with the manual removal of visible tartar (Figure 4). Accordingly, before any clinical intervention, antimicrobial therapy was performed using 0.12% chlorhexidine followed by the manual removal of tartar. During the mechanical removal of dental calculus, the dental health team used a high-powered suction system to aspirate the tartar fragments detached by manual scraping. This procedure was selected because of the risk of inhaling fragments; thus, one aspirator was placed in the oral cavity, and another was placed in the tracheostomy access.

At the end of the session, 2% chlorhexidine gel was applied with a toothbrush to drastically reduce the bacterial load in the oral cavity. A 2-month interval was determined for repetition of the procedure. Moreover, the family was encouraged to perform adequate mechanical control of the biofilm with the aid of a mouth opener, considering the difficulty of the patient in opening the mouth.

The second point of attention towards this patient considering the established protocol was the radiographic evaluation and monitoring of the balance between rhizolysis and rhizogenesis of the deciduous and permanent teeth, the need to perform extractions after a marked degree of rhizolysis of the deciduous teeth was observed, and the assessment of Nolla's developmental stage of the permanent teeth to avoid any chance of aspirating these teeth.

The home-based treatment involved the active participation of the mother in performing oral brushing for the patient using only a toothbrush, with the aim of disrupting the biofilm without the use of toothpaste. We did not recommend the use of toothpaste because it could be aspirated and cause future pulmonary complications. The parents were instructed to apply gauze moistened with 0.12% chlorhexidine 1 week per month.

This protocol was implemented at the initiation of patient treatment. Occasionally, povidone-iodine (PVP-I) was used in alternation with chlorhexidine, as the patient experienced no allergic reactions to iodine.

The patient is still being monitored after an 8-year follow-up period. The complications associated with periodontal disease decreased, and since the initiation of treatment, the patient has not needed to be hospitalized because of chronic lung disease.

In 2013, when the patient acquired permanent dentition and considering the periodontal changes characteristic of puberty, the focus of treatment shifted to the control of periodontal disease through the establishment of monthly consultations. The patient is currently at this stage of treatment.

## 3. Discussion

This case report describes the manifestations of Fraser syndrome, including cryptophthalmia, which is an important complication in this syndrome but is not always present.

Certain authors claim that one minor and two major diagnostic criteria or one major and at least four minor diagnostic criteria are necessary for the correct diagnosis of the disease [1]. Major criteria include cryptophthalmia, syndactyly, genital anomalies, and sharing the disease with a sibling. Minor criteria include congenital malformation of the nose, ear, and larynx, lip and palate cleft, skeletal defects, umbilical hernia, renal agenesis, and mental retardation. In the case reported, the patient had genital anomalies as a major criterion and congenital malformation of the ear and larynx, umbilical hernia, renal agenesis, and mental retardation as minor criteria.

Manifestations associated with Fraser syndrome include delayed dental development, prolonged retention of deciduous teeth, agenesis of second premolars, microdontia of deciduous molars, and formation of a broad zone of fibrous tissue in the vestibular mucosa [13]. None of these characteristics were observed in the patient evaluated.

The formation of calcified biofilm in the gingival area was due to the lack of mechanical removal of biofilm, which accumulated on the tooth surface over time. Certain systemic diseases cause biofilm to accumulate even further, warranting the use of drugs that reduce salivary flow [14]. The development of dental biofilm and consequentely bacterial growth in these patients can be potentializated, once they have motor skills difficulties to desorganize biofilms.

In the case reported herein, the difficulty and/or impossibility of self-care owing to the patient's mental retardation limited access to the oral cavity, promoting the formation of biofilm [15–17].

Among the systemic diseases, respiratory diseases are reported to be more closely associated with periodontal diseases. Previous studies indicated that periodontal diseases may influence the course of respiratory infections, especially pneumonia [18].

In this study, the removal of supragingival calculus and the decrease of bacterial load with chemotherapeutic agents reduced the bouts of chronic lung disease. This outcome may have resulted from the close association between the bacteria present in the oral cavity and the bacteria causing pneumonia and resulted in the effective decrease in hospitalization events.

Chlorhexidine is one of the most thoroughly studied antimicrobial agents and the most potent. It is highly effective and is generally used as a gold standard in dentistry for antimicrobial control; it is chemically classified as a bis-guanidine, with hydrophilic and hydrophobic properties [19].

Chlorhexidine has a broad antimicrobial spectrum and high substantivity, and it is safe and effective [20]. Initially, chlorhexidine molecules are adsorbed on the bacterial cell wall, where their positive charge interacts with negatively charged molecules present on the bacterial membrane surfaces, resulting in increased permeability of the bacterial membrane, entry of molecules into the cytoplasm, and the consequent disruption of the cell membrane and leakage of intracellular components [21]. Furthermore, the activity of chlorhexidine involves the impairment of glucose intake and lactic acid metabolism in* Streptococcus mutans* and decreased proteolytic activity in* Porphyromonas gingivalis* [22]. PVP-I was selected as an antimicrobial agent in this protocol because of its broad antimicrobial spectrum against Gram-positive and Gram-negative bacteria, fungi, mycobacteria, chlamydia, viruses, and protozoa. The antimicrobial activity of PVP-I is the result of strong oxidizing effects on amino groups (-NH), thiol (-SH), and phenolic hydroxy groups (-OH) in amino acids and nucleotides. Furthermore, PVP-I reacts strongly with double bonds in unsaturated fatty acids of the cell wall and organelle membranes [23].

We also decided to intercalate chlorhexidine with PVP-I to prevent microbial resistance.

The patient is currently in adolescence, and attention must be redoubled because hormonal changes during puberty are a predisposing factor for periodontal disease, as hormones can target the circulatory system, causing increased vascular permeability and gingival bleeding [24].

During adolescence, the colonization of the body by microorganisms changes because of the increased availability of sex hormones, and the load of biofilm-producing Gram-negative bacteria increases during this phase [25].

Increased hormone levels, particularly estrogen and progesterone, trigger an exacerbated response of the gingival tissue to the presence of the biofilm [26].

As previously reported [25], there is a direct association between increased levels of these two hormones and the increased number of anaerobic microorganisms, which play an important role in the onset and progression of periodontal disease by decreasing the phagocytic capacity of polymorphonuclear (PMN) leukocytes and increasing the secretion of interleukin-1b (IL-1b) [27]. In addition, these sex hormones increase vascular permeability and promote the interaction of proteolytic enzymes with interleukin-6 (IL-6), a mediator of inflammation [27].

Sex hormones modulate the production of cytokines. In this respect, progesterone decreases the production of IL-6 in gingival fibroblasts to 50% of the IL-6 levels observed when the levels of this hormone are normal [28] which is not the case for this patient.

The concomitant production of progesterone and estrogen can modulate the immune response through antigen expression and presentation, cytokine production, expression of apoptotic factors, and cell death. Progesterone stimulates the production of inflammatory mediators, for example, prostaglandin E2, and increases the number and chemotaxis of PMN leukocytes in the gingival sulcus. By contrast, estradiol decreases chemotaxis [27–29]. These data substantiate our current concerns for the carriers of Fraser syndrome about possible periodontal diseases to which they are exposed during adolescence and puberty.

The developmental period of T. H. O. and his hormonal and immune profile may affect his oral and overall health, particularly the occurrence of recurrent episodes of pneumonia. Therefore, at this stage of treatment, we intend to clinically control periodontal disease in view of infection complications, as immune factors cannot be modified through the continuous monitoring of the patient.

Despite the few case reports on Fraser syndrome in the specialized literature, the data available suggest an association between dental plaque, periodontal disease, and chronic lung disease. Therefore, the establishment of oral health protocols with a goal of reducing biofilm is essential to improve the health of these patients from the systemic point of view. In this case report, after the periodontal treatment with the clinical use of 2% chlorhexidine and PVP-I and the domiciliary use of 0.12% chlorhexidine, the complications associated with periodontal disease decreased, and since the initiation of treatment, the patient has not needed to be hospitalized because of chronic lung disease.

## 4. Conclusions

Despite the few case reports on Fraser syndrome in the specialized literature, the data available suggest an association between dental plaque, periodontal disease, and chronic lung disease. Therefore, the establishment of oral health protocols with a goal of reducing biofilm is essential to improve the health of these patients from the systemic point of view. In this case report, after the periodontal treatment with the clinical use of 2% chlorhexidine and PVP-I and the domiciliary use of 0.12% chlorhexidine, the complications associated with periodontal disease decreased, and since the initiation of treatment, the patient has not needed to be hospitalized because of chronic lung disease.

Based on the oral complications in this syndrome, the dentist should promote the patient's oral health and a good overall quality of life, as oral health is closely associated with overall health and as diseases in general, including oral diseases, cause biological impairments and the systemic dissemination of infections.
